# Dislocation of Femur Reduced on Sixty-eighth Day, by Jarvis’s Surgical Adjuster

**Published:** 1851-03

**Authors:** Josiah Crosby

**Affiliations:** Manchester, U. S.


*Dislocation of Femur reduced on sixty-eighth day, by Jarvis's Surgical Adjuster.* By Dr. Josiah Crosby, of Manchester, U. S.—On the 16th of January, 1849, Mr. P—, æt. 56, muscular, weighing about 180 pounds, of intemperate habits, was thrown from a sleigh, and dislocated his left hip. Six weeks passed before the nature of the injury was understood, and more than three weeks afterwards before any attempt was made to reduce it.

On the sixty-eighth day of the dislocation the author saw the patient for the first time—found him lying in bed with all the diagnostic signs of dislocation of the head of the femur upwards and backwards; limb below the natural temperature, purple, œdematous, and shortened from three to four inches. Although the case seemed almost hopeless, it was agreed that an effort at reduction should be made, and *Jarvis's Adjuster* was accordingly applied. Before extension was commenced, an attempt was made to bring the patient under the influence of chloroform, but it was soon abandoned on account of violent spasms which were produced by it, and the operation was done without the aid of this powerful agent. The extension was continued, gradually increasing the power for nearly twenty-four hours, when by a little rotation of the limb, the head of the bone was brought into the socket with a snap audible to the bystanders.

The hand of an assistant was placed on the trochanter while flexion and slight rotation were given to the limb, to see if the leg would remain at full length and in the right position. Everything proving satisfactory, the patient was laid in bed, and a bandage applied about the hips to guard against accidents from motion.

Dr. C. remarked that in the Report on Surgery to the National Medical Association for 1849, the chairman of that committee gives an unfavorable opinion for himself and Prof. Pope of the St. Louis University, in regard to the "Adjuster," but that no statistics were given on which this opinion is founded. He was of opinion that an instrument of so much power and of so varied application deserves a fair trial, and that by this test its character should be fixed.—*Ranking's Abstract.*

